# High-speed quantum networking by ship

**DOI:** 10.1038/srep36163

**Published:** 2016-11-02

**Authors:** Simon J. Devitt, Andrew D. Greentree, Ashley M. Stephens, Rodney Van Meter

**Affiliations:** 1 Center for Emergent Matter Science, RIKEN, Wakoshi, Saitama 315-0198, Japan; 2 Australian Research Council Centre of Excellence for Nanoscale BioPhotonics, and Chemical and Quantum Physics, School of Science, RMIT University, Melbourne 3001, Australia; 3 National Institute of Informatics, 2-1-2 Hitotsubashi, Chiyoda-ku, Tokyo 101-8430, Japan; 4 Faculty of Environment and Information Studies, Keio University, Fujisawa, Kanagawa 252-0882, Japan

## Abstract

Networked entanglement is an essential component for a plethora of quantum computation and communication protocols. Direct transmission of quantum signals over long distances is prevented by fibre attenuation and the no-cloning theorem, motivating the development of quantum repeaters, designed to purify entanglement, extending its range. Quantum repeaters have been demonstrated over short distances, but error-corrected, global repeater networks with high bandwidth require new technology. Here we show that error corrected quantum memories installed in cargo containers and carried by ship can provide a exible connection between local networks, enabling low-latency, high-fidelity quantum communication across global distances at higher bandwidths than previously proposed. With demonstrations of technology with sufficient fidelity to enable topological error-correction, implementation of the quantum memories is within reach, and bandwidth increases with improvements in fabrication. Our approach to quantum networking avoids technological restrictions of repeater deployment, providing an alternate path to a worldwide Quantum Internet.

Quantum communication will strengthen the security of cryptographic systems and decision-making algorithms^1^,^2^,^3^,^4^, support secure client-server quantum computation^5^, and improve the sensitivity of scientific instruments^6^,^7^,^8^. An effective global quantum network is ultimately required to support such applications^9^,^10^,^11^ and significant research has been conducted to realise such networks^12^,^13^,^14^,^15^. The most common method is the transmission of encoded optical signals along traditional fibre connections between more localised networks.

Photons are traditionally proposed for the establishment of quantum entanglement between stationary quantum systems over moderate distances. Over longer distances, entanglement purification and entanglement swapping connecting a path comprised of shorter links will mitigate the exponential attenuation loss, the no-cloning theorem^16^ and effects of imperfect devices^12^. The repeat-until-success nature of these techniques allows the rate of entanglement generation to decrease polynomially with increasing distance, with the fidelity of entanglement limited by the accuracy of the quantum gates operating in the repeaters. While experimental demonstration of short-range quantum communications has been effective^17^,^18^, long range repeater networks require the incorporation of fault-tolerant error correction methods, and numerous designs have been proposed^13^,^14^,^15^,^19^,^20^,^21^,^22^. These designs do not offer bandwidth higher than about a MHz and require dense repeater arrays. A global network constructed in this way would require high-power, low-temperature quantum devices with active control deployed in very hostile environments. While the deployment of satellite technology may mitigate this problem^23^, a fully error corrected, global system has still not been developed. Consequently, no known technology meets the stringent requirements for global deployment. In this work we present an alternative method that could be used to augment networks based on traditional repeaters and satellite technology. This approach can possibly solve crucial issues related to a global network in a way that remains compatible with other well known networking schemes.

Despite the capabilities of modern classical networks, it is still routine to transfer classical information stored in removable media, an approach known as *sneakernet*. Here we adapt this approach to quantum information, introducing a new network mechanism for the establishment of quantum entanglement over long distances based on the transport of error-corrected quantum memories^24^. Long-distance information transport involves significant latency, but establishing entanglement involves no exchange of users’ data, meaning that this latency is irrelevant to network users. Indeed the fact that entanglement distribution can occur without the exchange of classical information leads immediately to Peres’ well-known ‘paradox’ of delayed-choice entanglement swapping^25^,^26^. The fact that classical information need not be exchanged to distribute entanglement means that quantum networks can be effectively zero latency, although of course any protocol that uses the distributed entanglement, for example, for quantum teleportation^27^, will be limited by the classical one-way information transfer speed. Quantum memories may be transported to locations where entanglement is required or to intermediate locations to facilitate entanglement swapping between traditional quantum repeater networks, enabling a complete network structure without the full deployment of physical links. Because transoceanic communication presents a particular challenge for quantum networking, we focus on the establishment of entanglement by ship.

This approach of creating a quantum sneakernet through physical transport of actively error corrected quantum memories will require the fabrication of more physical qubits in the overall network, but has the possibility of significantly mitigating issues related to deployment of a quantum network. The most effective quantum repeater designs for entanglement distribution (so called 3rd generation repeaters) employ full error correction protocols and consequently require close separation (within of order 10 km) due to the need for low loss connections^13^,^15^. Each repeater unit is effectively a mini-quantum computer, requiring infrastructure technologies such as cryogenic cooling and vacuum systems, extensive classical control (for both qubit manipulations and error-correction/network operations) and non-trivial power requirements. The deployment of such devices, every 10 km, across an ocean constitutes an enormous engineering challenge. Conversely, a sneakernet approach allows us to convert this significant hurdle in deployment to the challenge of making mini-quantum computers amenable to physical transport. This allows us to design memory units that can be serviced (or replaced) at regular intervals with minimal impact on the overall network or incremental upgrades to the entire system as technology becomes denser and/or cheaper. This sneakernet method of quantum networking provides an alternate pathway that could augment traditional repeater and satellite based systems where the issue of network deployment and servicing in hostile environments becomes extremely hard or where entanglement nodes are required on mobile platforms not accessible by a traditional repeater node.

Our approach requires quantum memories with an effective coherence time of months, sufficient for transport along any traditional shipping channel. Because an error-corrected quantum memory is based on the same system architecture as a large-scale quantum computer, technology currently in development will satisfy our needs^28^,^29^,^30^. In particular, implementations of qubits based on several physical systems—including superconducting circuits and trapped ions—are nearing the accuracy threshold required for topological error correction to become effective^31^. Once this threshold is exceeded, it will be possible to arbitrarily lengthen the effective coherence time of an error-corrected quantum memory with a poly-logarithmic qubit overhead. However, this new mechanism also requires quantum memories that are compatible with storage and transport in a shipping container, including a stable power source, ultra-high vacuum or refrigeration systems to maintain appropriate operating conditions, and classical-control infrastructure to perform error correction and decoding^32^. As yet, there are no implementations designed with this degree of portability in mind. However, keeping in mind these engineering constraints, we will, for concreteness, consider a potential candidate system: an implementation based on negatively charged nitrogen vacancy (NV^−^) centres in diamond, which may be integrated in dense arrays and are optically accessible at a temperature of 4 K^30^,^33^.

The active nature of the quantum memory is designed to correct traditional errors arising from environmental decoherence and imperfect control. However, mobile quantum memories may be subject to additional errors arising from physical movement. These additional errors can arise from changes in, for example, the local magnetic field of the earth or from motional instability of the actual qubits. Most physical systems currently considered for large-scale quantum memories are immune from motional instability ether because they are etched circuits (superconductors, linear optics) or because they consist of atoms locked into a physical lattice (NV-diamond, silicon). Changes in global fields are not expected to cause significant problems for physical transport of these devices because global fields will vary on time scales that are far slower than the internal error correction utilised by each unit. Physical transport does introduce “catastrophic” failure channels (for example, physically losing the memory unit), but these failure channels do not impact the analysis presented in this work.

Each quantum memory consists of a two-dimensional array of optical cavities with a mean linear spacing of 0.66 mm, each containing a single NV^−^ centre comprising a spin-half N^15^ nucleus and a spin-one electron^30^,^33^. Each nuclear spin represents a single qubit, and all operations (initialisation, readout, and interactions between neighbouring qubits) are achieved by hyperfine coupling to the electron spins and dipole-induced transparency in an external optical field. We assume that these operations are fixed to a 3.5 *μ*s clock time and occur with an independent depolarising error rate of 0.1%, per gate^30^. Coherent control of spins in diamond has already been demonstrated with an error rate below 1%, indicating that this target may be achievable in the near future^34^. Each quantum memory stores a single logical qubit encoded in the surface code, although other codes may also be suitable. The error-correction protocol involves the continuous execution of physical quantum circuits to determine the error syndrome. This information undergoes local classical processing to detect and correct errors introduced by decoherence, coupling inefficiency, and other sources, thereby preserving the state of the logical qubit. To determine the effective coherence time of the quantum memories, we undertook numerical simulations of the error-correction protocol for small arrays. (see the supplementary material). Extrapolating to large arrays, we find that in this system, a quantum memory of approximately 4200 qubits enables storage of a logical qubit for approximately 40 days with a logical error rate of 10^−10^ [See the methods section].

Quantum memories are installed in Twenty-foot Equivalent Unit (TEU) containers, the standard shipping unit with an internal volume of 40 m^3^. We assume that 1 m^3^ is occupied by quantum memories and the remaining 39 m^3^ is reserved for power, refrigeration, and control infrastructure. Each of these units is the quantum equivalent of a memorystick. More efficient protocols may be achievable under different assumptions, but for simplicity we assume a single, dedicated Very Large Container Ship (VLCS)-class container ship with a capacity of 10^4^ TEU. We consider a shipping channel between Japan and the United States, with freight terminals acting as primary network nodes for traditional repeater networks deployed in each country, as shown in Fig. 1. Allowing for local transport and maintenance, this channel requires a one-way transport time of 20 days [http://www.joc.com/sailings, “Journal of Commerce, sailing schedules”, (2016) (Date of access:01/07/2014)].

Table 1 reports the effective transpacific bandwidth of the network mechanism. Our results compare quantum memories based on NV^−^ centres in diamond to quantum memories based on a range of other qubit implementations^28^,^29^,^30^,^35^,^36^,^37^,^38^,^39^ illustrating the dependence of the bandwidth on the underlying physical parameters. Each implementation involves a unique set of technological challenges and our results are predicated on the development of portable systems incorporating high-speed external interfaces to facilitate lattice-surgery operations between logical qubits^40^. Nevertheless, bandwidth in excess of 1 THz is feasible under realistic physical assumptions, exceeding even the fastest proposals for traditional repeater networks. Our results assume a single container ship, but the total bandwidth scales linearly with the total freight capacity, allowing for incremental investment in infrastructure rather than the overhaul of thousands of kilometres of undersea cables. Furthermore, adding network nodes involves transport to additional locations rather than investment in wider area infrastructure, and can be done with minimal planning and only a few weeks lead time.

In addition to expanding the reach of traditional repeater networks, our mechanism may be used to hybridise networks with different operating regimes. For example, quantum memories may be used to interconvert between repeater networks with different networking protocols, qubit implementations, or operating rates^40^. Quantum memories embedded in portable devices may allow mobile nodes to connect to static networks.

Our assumptions regarding the amount of space within each unit reserved for the actual qubits and necessary control infrastructure are somewhat arbitrary due to the unknown nature of how classical control technology will develop. An underlying assumption of this work is that a high speed, high fidelity quantum network is only describable in a future where large-scale quantum technology exists. While research related to the classical control of active quantum memories is in its infancy^32^,^42^,^43^, initial efforts in commercialising quantum technology suggests that our estimates are not overly optimistic. D-WAVE provides an example of a commercial package that requires cryogenic cooling, power and classical control [The DWAVE two system (www.dwavesys.com/d-wave-two-system) utilises a closed cycle dilution refrigerator, magnetic shielding, vacuum system and classical control in a size approximately the same as a TEU container].

As quantum technology advances, a network architecture based on the transport of reliable quantum memories could enable fundamental tests of quantum mechanics over long distances and then increasingly sophisticated applications ranging from quantum cryptography to distributed quantum computing. Our mechanism has the flexibility to service these applications as they develop, in addition to complementing traditional repeater networks as they are deployed. Eventually, once quantum computers are commonplace, entanglement will be the fungible resource that enables a vast range of distributed applications. The quantum sneakernet is a mechanism that could feasibly underpin an entanglement-based economy of this kind, connecting users of local quantum networks to a global Quantum Internet.

## Methods

### Capacity of a memory unit

**Figure 2:**
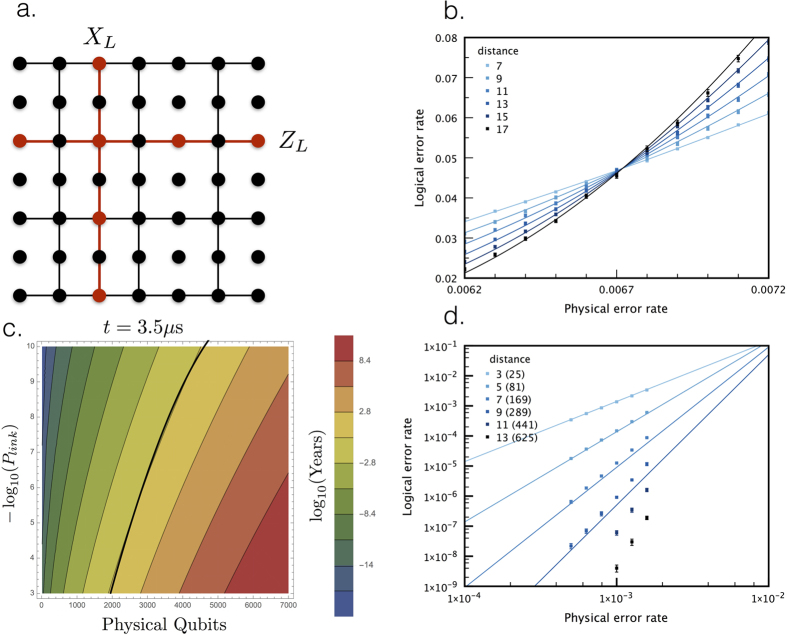
Properties of the planar code. The memory unit is a two-dimensional nearest neighbour array of qubits encoded with the topological planar code^24^. (**a**) Structure of the planar code with the single qubit *X*_*L*_ and *Z*_*L*_ operators. (Gridlines do not represent entanglement bonds.) (**b**) Logical error rate as a function of physical error rate for different code distances. Simulations confirm that the fault-tolerant threshold of the planar code lies at *p* ≈ 0.7% under a standard error model. (**c**) Memory time as a function of *N* and *P*_*link*_ for a device that has a *t* = 3.5 *μ*s physical gate time and *p* = 0.1%. Along the heavy black contour line, an *N*-qubit memory unit can maintain sufficiently high coherence to achieve the selected *P*_link_ after one year of storage time. (**d**) Per-error correction cycle logical error rate *P*_*L*_ as a function of physical error rate *p* for different code distances. Numbers in parentheses are total physical qubits per logical qubit at that code distance.

Illustrated in Fig. 2 is the structure and performance of a memory unit. The device encodes a single logical qubit of memory [Fig. 2a]. The logical Pauli operators are chains of physical *X* and *Z* operations that connect the top and bottom (logical *X*) or the left and right (logical *Z*) edges of the lattice. Through simulation, we numerically determine both the fault-tolerant threshold for the memory unit [Fig. 2b] and the expected failure rate as a function of QEC strength at a fixed physical error rate, *p* [Fig. 2d]. From the behaviour of the code for low values of the physical error rate, *p*, we can estimate the probability that a memory unit fails during one error correction cycle, *P*_*L*_, as a function of the distance of the topological planar code, *d* (an error correction cycle requires *d* rounds of error correction). For an operational device, we assume the error rate for each physical gate in the quantum memory is, *p*. The functional form for the failure of the code is given by,


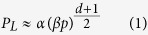


We use the data from Fig. 2, which simulates a full *d* rounds of error correction to estimate *α* ≈ 0.3 and *β* ≈ 70. The total number of physical qubits in the memory unit is *N* = (2*d* − 1)^2^ and the total time of a memory correction cycle is *T*_corr_ = 6*td*, where *t* is the operational time of a *physical* quantum gate (initialisation, measurement or CNOT), the factor of 6 comes from the six elementary gates necessary to perform syndrome extraction in the topological code, and we require *d* rounds of error correction to correct for measurement errors.

The total memory time of the unit, *T*_mem_, is related to the per-error correction cycle failure probability, *P*_*L*_, and the chosen permissible infidelity of the final entangled link, *P*_link_ = 1 − *F*, where *F* is the link fidelity (between memory units),


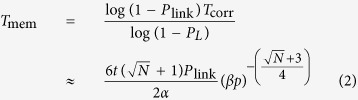


Figure 2b shows the memory time for a device that has a *t* = 3.5 *μ*s physical gate time (appropriate for optically coupled NV^−^ 30), as a function of total number of physical qubits, *N*, and desired final link infidelity, *P*_link_. The contour where the memory unit can maintain coherence for one year and achieve the desired link fidelity is plotted with a heavy line. Similar plots can be easily obtained from Eqn. 2 for different physical gate times, *t*. As it is assumed that the physical system is at a fixed physical error rate *p* = 0.1% (or *p* = 0.001%) regardless of the intrinsic gate speed of the system, *t*, memory times will increase with slower systems. Ion trap computers will have a *longer* memory time than donor-based systems as we assume *both* technologies can achieve a *p* = 0.1% (0.001%) error rate on all fundamental gates. For a *N* = 4225 qubit memory unit for the optically coupled NV^−^ system. Taking 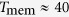 days as our target memory time, we find the link infidelity achievable is approximately *P*_link_ ≈ 10^−10^ (We assume a 40 day storage time for a 20 day transit time to account for preparing and consuming the Bell states at the source or destination. This in practice can be reduced to 20 days by strategic choices of which Bell states to prepare and consume at given points in time, but this does not significantly change the size of each memorystick). For all other technologies we recalculate the size of the memory unit to achieve the same infidelity and memory time given the physical gate time, *t*, and the physical error rate, *p*.

### Lattice surgery operations

The planar code (and all toric code derivatives) allows a logical two-qubit CNOT gate to be executed as a transversal operation using individual CNOT gates applied between corresponding qubits in each memory unit. While fault tolerant, this method may be difficult to implement due to the difficulty of ensuring that each qubit in the 2D memory cell can interact with the corresponding qubit in another cell. A different approach, called *lattice surgery*, partially solves this problem by realising a fault-tolerant CNOT gate between two memory units by only using interaction between qubits along an edge of each memory unit.

Lattice surgery works by merging two separate lattices, each containing a single logical qubit encoded in the planar code, into a single oblong lattice, then splitting up this single planar code again. The merging operation is done by matching the edges of two distinct logical qubits and measuring code stabilisers spanning the lattice cells. This effectively reduces a two qubit encoded system to a single encoded qubit. This merging takes the state 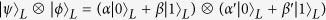 to 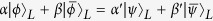, where 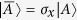. The measurement of the stabilisers to perform a merge must occur *d* times, where *d* is the effective code distance of each planar code. This protects against faulty qubit measurements for each stabiliser measurement. Given that the quantum circuit required to measure the stabilisers for the planar code requires 6 physical gates, the merge operation requires a time of *T* = 6*td*, for physical gate times *t*.

The splitting operation is executed by physically measuring the qubits along the merged edge to divide the single lattice back into two individual lattices. The effect of a split operation is to take the single logical state encoded in the joint lattice, 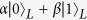 to the two-qubit state, 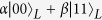. Once again to protect against measurement errors, error correction of both lattices must be run for a total of *d* cycles, requiring a total time of *T* = 6*td* for the split operation.

Given these transformations, we can construct a Bell state between two encoded memory units by initialising a *d* × *d* lattice holding a logical qubit in one memory unit in the 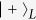 state and a logical qubit in the other memory unit in the 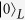 state, merge the edges of the lattices across the optical interface between units to form a single state 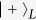 in a 2*d* × *d* distance lattice, and then split them again to create the state 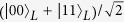, with one logical qubit held in each memory unit. This state can be manipulated through transversal Hadamard operations on each memory cell and/or *X*_*L*_ and *Z*_*L*_ to any of the three other Bell states in either the *X*- or *Z*-basis. The total time for the split/merge operation will be 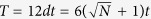 for a physical gate time of *t* and a memory cell containing *N* qubits. For the NV^−^ design described above, *t* = 3.5 *μ*s and for *N* = 4225, *T* ≈ 1.4 ms.

### Network operational procedures

Our network protocol ensures that the effective transoceanic bandwidth is limited only by the freight capacity and the transport time. A total of seven shipping containers are utilised for each “online” pair. Two units are permanently located at each shipping terminal. Three mobile units rotate locations; at any point in time, one is at each terminal (*A* or *B*) and the third is aboard ship. The protocol is separated into three phases, which operate sequentially in each direction. In the first phase, each mobile logical qubit is entangled with a stationary logical qubit at the origin to establish logical Bell pairs. In the second phase, one logical qubit in each logical Bell pair is transported from the origin to the terminus, undergoing continuous error correction. In the third phase, the logical qubits in the logical Bell pairs are entangled with additional logical qubits at the origin and the terminus and then measured to provide end-to-end entanglement swapping at the logical level.

For example, consider the case in which a stationary unit sitting at terminal *A* is entangled with a mobile memory unit at terminal *B*, and this pair is used as the online pair for supplying terminal-to-terminal entanglement to other parts of the network. After the remote entanglement supply in the mobile unit is exhausted, it will be re-entangled with another stationary memory unit at its current location. A second mobile memory unit is aboard ship, entangled with a stationary memory unit at the shipping terminal from which it departed, carrying entanglement from *B* to *A*. A third mobile memory unit at *A* is creating entanglement with a stationary local partner in preparation for shipping. This ensures that ships are never transporting inactive (unentangled) memory units.

The fixed constraints on the bandwidth of a link are latency of the ship, the capacity of a memory unit, and the physical gate time, from which we can derive additional operational procedures and hardware development goals. The fixed 20-day transit time for the Japan-U.S. link serves as an upper bound for completing the entanglement of a mobile memory unit with a stationary memory unit, and for consuming the entanglement after shipping. The *T* = 1.4 ms logical Bell pair creation time above arises from a surface code distance *d* = 33 and gate operation time *t* = 3.5 *μ*s, for an NV^−^ optical implementation^30^, and assumes that inter-container operations can be executed at the same rate as operations local to each memory unit. (For the optical NV^−^ system, this is a reasonable assumption, because the intra-container operations use the same physical mechanism as the inter-container operations.) At this operation rate, the entanglement of a memorystick containing approximately 12.7 KEb (Kilo-Entangled-bit) in the NV^−^ optical approach is created or consumed in 18 s, and at full rate an entire shipload of entanglement would be consumed in a little over two days. Thus, inter-container operations may be 10× slower without impacting the performance even if only one container at a time out of an entire shipload is used online. To avoid long periods of unavailability of the network, it may be desirable to limit the rate at which entanglement is provided to applications to one-tenth of the achievable physical rate.

The denser memory subsystems, differing physical gate times, and varying code distances for other options in Table 1 will result in different demands on the inter-container interfaces. Because of the generic nature of the created entanglement, slow inter-container interfaces can be compensated for by having more than a single container online.

To achieve the performance in the right hand column of Table 1, the containers on board ship must collectively provide that much bandwidth. For the first three entries in the table, we expect that having a single container online with a simple serial interface will be sufficient. For the higher data rates, parallelism can be used both at the container interface and by having all 10^4^ containers online at the same time. Each container must provide 10^6^–10^8^ logical entanglements/second. These entanglement operations may be multiplexed through a single connection, or more likely carried in parallel through a parallel waveguide bundle. Even so, achieving this level of performance will require advances in optical entanglement methods for the physical technologies. Thus, this table serves as a performance target for experimental work.

## Additional Information

**How to cite this article**: Devitt, S. J. *et al.* High-speed quantum networking by ship. *Sci. Rep.* **6**, 36163; doi: 10.1038/srep36163 (2016).

**Publisher’s note:** Springer Nature remains neutral with regard to jurisdictional claims in published maps and institutional affiliations.

## Figures and Tables

**Figure 1:**
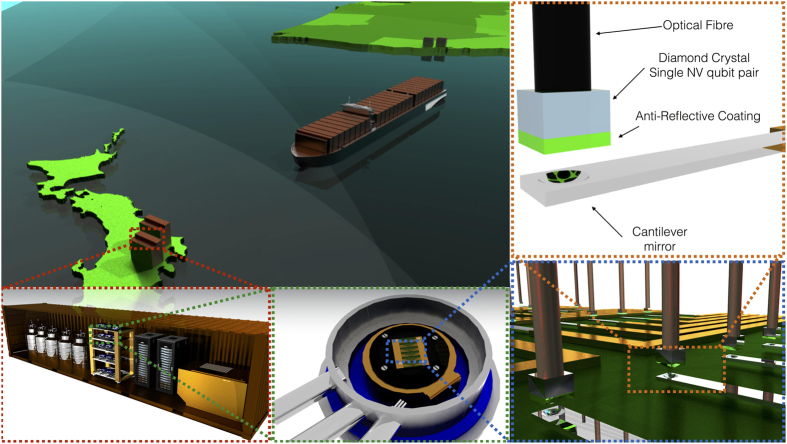
Physical transport protocol for a transpacific sneakernet using a single ship. The transpacific connection shows the location of memory stick units both on shore in the U.S. and Japan and in transit aboard a VLCS-class ship. Each cargo container (memorystick) contains the actual memory units as well as any required control, cooling and power infrastructure. Each memory unit (for the specific hardware model of optically connected NV^−^ qubits^30^) consists of an array of diamond crystals within adjustable single sided cavities which are optically connected to perform individual qubit/qubit interactions^33^.

**Table 1:** Effective bandwidth of a transoceanic link.

Implementation	Qubit pitch (m)	Gate time (s)	Physical error rate	(*d*, *N*)	Memorystick capacity	Bandwidth (Hz)
NV^−^ (optical)	6.6 × 10^−4^ 33	3.5 × 10^−6^ 30	1 × 10^−3^	(33, 4225)	12.7 KEb	7.3 × 10^1^
Trapped ions	1.5 × 10^−3^ 36	1.0 × 10^−4^ 35	1 × 10^−5^	(11, 441)	32 KEb	1.9 × 10^2^
Transmons	3.0 × 10^−4^ 39	4.0 × 10^−8^ 31	1 × 10^−5^	(13, 625)	2.4 MEb	1.4 × 10^4^
Quantum dots	1.0 × 10^−6^ 28	3.2 × 10^−8^ 28	1 × 10^−3^	(36, 5041)	2.8 TEb	1.6 × 10^10^
NV^−^	3.0 × 10^−7^ 29	1.0 × 10^−3^ 29	1 × 10^−3^	(29, 3249)	200 TEb	1.6 × 10^12^
silicon	2.0 × 10^−7^ 38	5.0 × 10^−8^ 37	1 × 10^−3^	(36, 5041)	350 TEb	2.0 × 10^12^

Effective bandwidth achieved using a single VLCS-class container ship transporting error-corrected quantum memories between Japan and the United States, estimated for a range of qubit implementations for a fixed infidelity of 1 − *F* = 10^−10^. For several implementations, bandwidth exceeds the fastest proposals for traditional repeater networks at far lower infidelities. Memorystick capacities (in Entangled bits, or Ebits, Eb) are estimated as 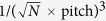 for a memory unit of *N* qubits given the operational gate time and error rate for a 40 day storage time when utilising 1 m^3^ of space within each container. Figures for physical error rates are development targets for production-use hardware. They are chosen assuming more experimentally mature technologies can achieve a physical error lower than less mature technologies.
